# Cost-effectiveness analysis of trastuzumab to treat HER2-positive advanced gastric cancer based on the randomised ToGA trial

**DOI:** 10.1038/bjc.2011.390

**Published:** 2011-09-29

**Authors:** T Shiroiwa, T Fukuda, K Shimozuma

**Affiliations:** 1 Department of Biomedical Sciences, College of Life Sciences, Ritsumeikan University, 1-1-1, Noji-higashi, Kusatsu, Shiga 525-8577, Japan; 2 Department of Health Economics and Epidemiology Research, School of Public Health, University of Tokyo, 7-3-1, Hongo, Bunkyo-ku, Tokyo 113-0033, Japan

**Keywords:** gastric cancer, cost effectiveness, trastuzumab, cisplatin, capecitabine

## Abstract

**Background:**

We performed a cost-effectiveness analysis of trastuzumab plus chemotherapy for human epidermal growth factor type-2 (HER2)-positive advanced gastric cancer (GC) based on data obtained from the Trastuzumab for Gastric Cancer (ToGA) trial from a Japanese perspective.

**Methods:**

The following Japanese and Korean populations of the ToGA trial were analysed to obtain mean overall and progression-free survival times: (1) all HER2-positive populations, (2) immunohistochemical (IHC) 2+/fluorescence *in situ* hybridisation (FISH)+ or IHC 3+ populations, and (3) IHC 3+ only population. The effect of trastuzumab treatment on mean survival time was estimated by fitting a Weibull parametric function. Costs were calculated from the perspective of health-care payer. Neither costs nor outcomes were discounted because of short life expectancy.

**Results:**

In the base-case analysis, the incremental cost-effectiveness ratio was (1) JPY 12 million (€110 000) per quality-adjusted life year (QALY) gained and JPY 8.9 million (€81 000) per life-year gained (LYG) for all HER2-positive populations, (2) JPY 9.1 million (€83 000) per QALY gained and JPY 6.6 million (€60 000) per LYG for the IHC 2+/FISH+ or IHC 3+ population, and (3) JPY 6.1 million (€55 000) per QALY gained and JPY 4.3 million (€39 000) per LYG for the IHC 3+ population.

**Conclusion:**

Trastuzumab treatment for IHC 3+ populations is cost effective. Our analysis can find a cost-effective subgroup when advanced GC is treated by trastuzumab.

Gastric cancer (GC) has the third highest age-standardised mortality among cancers worldwide, trailing lung and breast cancer in 2008 (Ferlay *et al*, 2010). Gastric cancer is especially prevalent in East Asia than in Europe and America, as mortality adjusted by age in East Asia is about twice the worldwide rate for both genders (19.8 in males worldwide *vs* 42.4 in East Asia, and 9.1 for females worldwide *vs* 18.3 in East Asia). Thus, GC remains an important public health concern.

Platinum-based regimens combined with fluorouracil (some regimens also add an anthracycline or doxetaxel) are widely used as first-line therapy for advanced GC. A meta-analysis of treatment effects shows that chemotherapy could prolong overall survival (OS) compared with best supportive care (hazard ratio (HR) 0.37; 95% confidence interval (95% CI) 0.24–0.55, *P*<0.001) (Wagner *et al*, 2010). The same analysis also indicated that combined therapy is more effective than is single therapy (HR 0.82; 95% CI 0.74–0.90, *P*=0.001). Randomised controlled trials (RCTs) also show that oral capecitabine could be substituted for infused fluorouracil, avoiding the necessity of infusion pumps and central venous catheterisation (Cunningham *et al*, 2008; Kang *et al*, 2009; Okines *et al*, 2009).

Recently, molecular targeted therapies such as monoclonal antibodies have been explored with chemotherapy. An example of this is trastuzumab (Herceptin). Trastuzumab is a humanised monoclonal antibody that selectively targets human epidermal growth factor type-2 (HER2) receptors (Slamon *et al*, 1987). Several RCTs have confirmed the usefulness of trastuzumab for patients with HER2-positive breast cancer in the metastatic (Slamon *et al*, 2001) and adjuvant setting (Piccart-Gebhart *et al*, 2005; Romond *et al*, 2005; Smith *et al*, 2007). The amplification of the *HER2* gene and overexpression of the HER2 protein are considered poor-prognosis factors and are observed among 20–30% of breast cancer patients.

The ‘Trastuzumab for Gastric Cancer’ (ToGA) trial was a phase III, open-label, RCT comparing trastuzumab with platinum-based chemotherapy against chemotherapy alone as a first-line treatment for advanced GC with HER2 overexpression or amplification (Bang *et al*, 2010). The ToGA trial showed that the addition of trastuzumab to platinum-based chemotherapy significantly improved OS. Median OS was 13.8 months in the trastuzumab with chemotherapy-treated group compared with 11.1 months in the control group (HR 0.74; 95% CI 0.60–0.91, *P*=0.0046). The ToGA trial also indicated that trastuzumab was more effective in treating patients with high HER2 expression than those with low expression. The HR of OS for patients with immunohistochemical (IHC) 2+/fluorescence *in situ* hybridisation (FISH)+ or IHC 3+ was 0.65 (95% CI 0.51–0.83) and the HR for those with IHC 3+ was 0.58 (95% CI 0.41–0.81), although the IHC2+/FISH+ and IHC3+ subgroups were *post hoc* defined groups. Although the IHC3+ subgroup was pre-planned, both were exploratory analyses.

Despite these benefits, the use of new molecular targeting drugs such as trastuzumab could increase the economic burden of treatment. Thus, it is important to consider whether additional costs justify the outcome. Such a consideration of cost effectiveness may be critical in deciding which patients are treated with trastuzumab.

## Materials and methods

### Framework of cost-effectiveness analysis

We performed a cost-effectiveness analysis of chemotherapy with or without trastuzumab as a first-line therapy for treating advanced GC in a Japanese health-care setting based on data obtained from the ToGA trial. Our analysis fundamentally followed the recommendations of the Panel on Cost-Effectiveness in Health and Medicine (Gold *et al*, 1996). Two clinical outcomes, namely quality-adjusted life year (QALY) gained and life year gained (LYG), as well as an estimate of overall costs, were used to calculate the incremental cost-effectiveness ratio (ICER). For this cost-effectiveness analysis, we took the perspective of the health-care payer, including only direct medical costs. Indirect costs such as work loss were not accounted for. As patients with advanced GC have OS times of ∼12 months only, neither costs nor clinical outcomes were discounted because of the short time horizon of the trials.

### Patients

**Figure 1:**
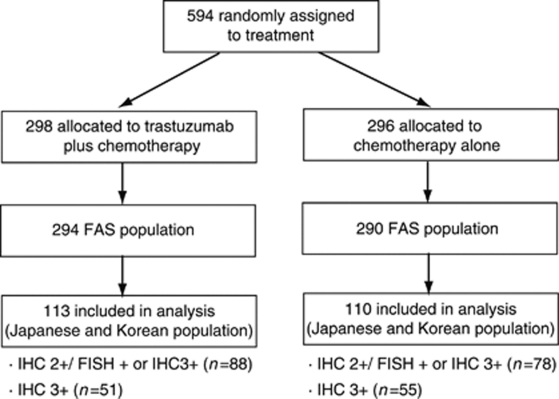
Schematic describing randomised patients.

In the ToGA trial, advanced GC patients were randomly assigned to receive trastuzumab with chemotherapy or chemotherapy alone. Our analysis was performed only in the Japanese and Korean full analysis set population (*n*=223). Cost-effectiveness analysis was performed for the following three populations: (1) all HER2-positive populations, (2) the IHC 2+/FISH+ or IHC 3+ population, and (3) the IHC 3+ population. The rationale for considering the data in this manner was that the ToGA trial showed that trastuzumab treatment was more effective in patients with higher HER2 protein expression. Figure 1 details the patients and Table 1 shows the characteristics of the population.

### Chemotherapy regimens

In the ToGA trial, advanced GC patients randomly received chemotherapy alone or chemotherapy with trastuzumab, as follows: (1) chemotherapy: capecitabine 1000 mg m^−2^ (twice a day for 14 days followed by a 1 week rest), or fluorouracil 800 mg m^−2^ per day (continuous intravenous infusion on days 1–5 of each cycle) and cisplatin 80 mg m^−2^ (on day 1 by intravenous infusion) every 3 weeks for 6 cycles and (2) trastuzumab: 8 mg kg^−1^ (on day 1 of the first cycle), followed by 6 mg kg^−1^ every 3 weeks until disease progression.

### Medical resource use and costs

Medical resource usage resulting from anti-cancer drug administration was estimated based on doses of medications used in the ToGA trial. The chemotherapy fee (including fees for HER2 testing, cardiac monitoring, in-patient and outpatient chemotherapeutic medications, blood testing, diagnostic imaging, and pharmaceuticals) was included according to the chemotherapy protocol. Pre-medication drugs before cisplatin administration (5-HT3 antagonists, corticosteroids, and diuretics) were also included. Costs of second-line and subsequent chemotherapy were considered according to the rate of anti-cancer drug use after progression in the ToGA trial (Table 2), excluding drugs under development and off-label drugs. Although 81% patients received second-line therapy, only 40% of the general population received it.

In the base-case analysis, adverse event (AE) costs were not considered because the frequency did not vary widely between groups (Bang *et al*, 2010). This parameter was subjected to sensitivity analysis.

Unit costs were calculated for the year 2010 based on medical fee schedules set out by National Health Insurance (NHI) (Jiho, 2010b) and NHI drug price list (Jiho, 2010a). The prices of anti-cancer drugs were (1) trastuzumab JPY 56 110 (€510) (150 mg vial) and JPY 23 992 (€220) (60 mg vial), (2) cisplatin JPY 13 513 (€120) (100 mg vial), JPY 7714 (€70) (60 mg vial) and JPY 3166 (€29) (10 mg vial), and (3) capecitabine JPY 350.5 (€3.2) (300 mg tablet) as of December 2010 based on an exchange rate of €1=JPY 110. Censored data were used to calculate mean cost per patient, according to the method of Lin *et al* (1997).

### Calculation of QALY

Let P_*i*_ be mean progression-free survival (PFS) of group *i*, O_*i*_ be mean OS of group *i*, U_p,i_ be utility score of PFS (group *i*), and U_d_ the utility score of disease progression. We calculated QALY gain of group *i* c by P_*i*_ × U_p,i_+ (O_*i*_−P_*i*_) × U_d_. Survival curves of PFS and OS (P_*i*_ and O_*i*_) were estimated by fitting a Weibull survival curve to the patient level data using maximum likelihood method. Mean PFS and OS were calculated by taking the area under respective survival curve.

Utility scores of PFS were 0.815 (trastuzumab group) and 0.797 (chemotherapy alone) from the EuroQoL (EQ-5D) responses of the ToGA trial calculated by Japanese scoring algorithm (Tsuchiya *et al*, 2002). These EQ-5D values were analysed based on a linear mixed model using the MIXED procedure in SAS 9.1 (SAS Institute, Cary, NC, USA). Within the TOGA trial, the EQ-5D was not administered after disease progression and therefore utility values from an alternative source was identified. Thus, in the base-case analysis, we used the score of 0.6 based on National Institute for Health and Clinical Excellence (NICE) technology appraisal 208 (NICE, 2010) (NICE used a score of 0.577 based on a previous appraisal of sunitinib for GIST). The impact of this value on the model results was assessed using sensitivity analysis.

### Sensitivity analysis

We initially performed sensitivity analyses on the costs of AEs and utility scores after progression, which potentially influence the results. The uncertainty was also evaluated, based on a bootstrap method (Efron and Tibshirani, 1994), repeating bootstrap re-sampling 10 000 times. Using these bootstrap results, we created acceptability curves (van Hout *et al*, 1994) depicting the percentage for which trastuzumab is cost effective in relation to the cost-effectiveness threshold.

## Results

### Results of base-case analysis

**Figure 2:**
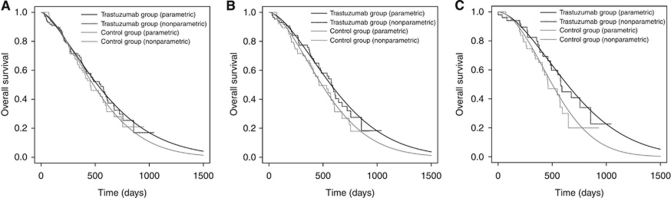
Survival curves. (**A**) All the HER2-positive population. (**B**) IHC 2+/FISH+ or IHC 3+ population. (**C**) IHC 3+ population.

Results of the cost-effectiveness analysis are shown in Table 3. Survival curves obtained from the Weibull regression and empirical Kaplan–Meier method are shown in Figure 2.

#### All HER2-positive populations

If trastuzumab was administered for all patients with HER2-positive advanced GC patients, the mean QALYs (life years) gained were 1.168 (1.671) in the trastuzumab with chemotherapy group *vs* 1.048 (1.489) in the chemotherapy-alone group. Thus, the difference in outcome is 0.134 QALYs gained (0.183 life years). Expected medical costs in the trastuzumab group were estimated to be JPY 2.9 million (€27 000) per patient, which corresponds to an increased cost of JPY 1.6 million (€15 000) per patient over chemotherapy alone (JPY 1.3 million (€12 000) per patient). The ICER of trastuzumab was JPY 12 million (€110 000) per QALY gained and JPY 8.9 million (€81 000) per LYG.

#### IHC 2+/FISH+ or IHC 3+ population

Mean survival gain was 1.238 QALYs (1.764 life years) per patient receiving trastuzumab and 1.056 QALYs (1.495 life years) per patient not treated with trastuzumab. The estimated cost of trastuzumab treatment was JPY 3.1 million (€28 000) *vs* JPY 1.3 million (€12 000) for chemotherapy alone. The ICER of using trastuzumab was JPY 9.1 million (€83 000) per QALY gained and JPY 6.6 million (€60 000) per LYG.

#### IHC 3+ population

Among the IHC 3+ population, the estimated incremental clinical outcomes with trastuzumab treatment are 0.326 QALYs (1.371 QALYs in trastuzumab with chemotherapy *vs* 1.060 QALYs with chemotherapy alone). Trastuzumab was estimated to improve life expectancy by 0.462 (1.935 LYG in trastuzumab with chemotherapy *vs* 1.473 LYG in chemotherapy alone). The cost of trastuzumab with chemotherapy was estimated to be JPY 3.3 million (€30 000) and that of chemotherapy alone to be JPY 1.4 million (€12 000). Trastuzumab treatment increases medical costs by JPY 2.0 million (€18 000) per patient. The ICER of trastuzumab was JPY 6.1 million (€55 000) per QALY gained and JPY 4.3 million (€39 000) per LYG, which is lower than all HER2-positive populations and IHC 2+/FISH+ or IHC 3+ population.

### Sensitivity analysis

The results of the sensitivity analysis for ‘costs of AE’ and ‘utility scores after progression’ are shown in Table 4. Results suggest that additional AE costs of trastuzumab did not greatly influence the ICER, utility scores after progression influence the ICER of trastuzumab. When utility scores were changed from 0.4 to 0.8, the ICER increased from JPY 10 million (€93 000) per QALY gained to 15 million (€140 000) per QALY gained in all HER2-positive populations, from JPY 7.7 million (€70 000) per QALY gained to 11 million (€100 000) per QALY gained in the IHC 2+/FISH+ or IHC 3+ population, and from JPY 5.1 million (€47 000) per QALY gained to 7.5 million (€68 000) per QALY gained in the IHC 3+ population.

**Figure 3:**
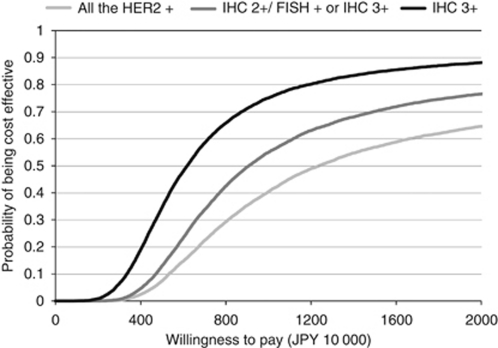
Acceptability curves.

The results of acceptability curves are shown in Figure 3. If the threshold of cost-effectiveness analysis is JPY 6.0 million (€55 000) per QALY gained, the probability that trastuzumab is cost effective was 14.7% for all HER2-positive populations, 23.4% for the IHC 2+/FISH+ or IHC 3+ population, and 47.9% for the IHC 3+ population. When the threshold increases to JPY 10 million (€91 000) per QALY gained, the probability of cost effectiveness was 40.4% for all HER2-positive populations, 54.9% for the IHC 2+/FISH+ or IHC 3+ population, and 75.1% for the IHC 3+ population.

## Discussion

Our cost-effectiveness analysis indicated that the ICER was (1) JPY 12 million (€110 000) per QALY gained and JPY 8.9 million (€81 000) per LYG for all HER2-positive populations, (2) JPY 9.1 million (€83 000) per QALY gained and JPY 6.6 million (€60 000) per LYG for the IHC 2+/FISH+ or IHC 3+ population, and (3) JPY 6.1 million (€55 000) per QALY gained and JPY 4.3 million (€39 000) per LYG for the IHC 3+ population. Sensitivity analysis showed that utility scores after progression, but not the costs of AEs, had the largest influence on the estimated cost-effectiveness of trastuzumab.

As yet, there has been only one other economic evaluation of trastuzumab for treating advanced GC, which is described by NICE of the United Kingdom (NICE, 2010). According to this report, the ICER of trastuzumab combined with epirubicin plus cisplatin and capecitabine lies between £63 100 and £71 500 per QALY gained for IHC 2+/FISH+ or IHC 3+ populations compared with chemotherapy alone. The NICE concluded that the ICER was not cost effective. However the ICER lies between £45 000 and £50 000 per QALY gained when the population is limited to HER2 (IHC 3+) population. After applying an ‘end-of-life’ guidance wherein estimated mean QALYs are considered nearly equal to life years (to weigh the value of end-of-life period), and NICE recommended the use of trastuzumab for only IHC 3+ population. The cost-effectiveness threshold for treatments considered under the ‘end-of-life’ guidance is higher, and the ICER for the IHC3+ subgroup as quoted above was considered acceptable.

The analysis by NICE was based on an indirect comparison, not based on the head-to-head results from the ToGA trial. However the concept of NICE's ‘end-of-life’ guidance is worthy of consideration. That is, should one QALY for life-threatened patients (e.g., cancer patients) be treated as equal to one QALY for patients who have good chances of recovery (e.g., those with the flu)? Although it is difficult to answer this question, such discussion was actually started in the value-based pricing in the United Kingdom (Department of Health, 2010). It may be important to consider not only cost per QALY but also cost per LYG in making decisions that reflect individual preferences more appropriately, especially in the area of oncology.

No consensus exists with regard to the threshold of acceptable cost per QALY ratios in Japan. In the United Kingdom, NICE uses a threshold range of £20 000 to £30 000 (approximately JPY 2.5 million to JPY 4.5 million) per QALY gained (NICE, 2008), although it is possible that a higher threshold is acceptable for treatments considered under the ‘end-of-life’ guidance. In the United States, USD 50 000 to USD 100 000 (JPY 4.5 million to JPY 9.0 million) per QALY gained is often used. A Japanese study on willingness to pay for one additional QALY suggests that JPY 5.0 million (€45 000) to JPY 6.0 million (€55 000) is appropriate as a threshold (Shiroiwa *et al*, 2010). Considering the results of our cost-effectiveness analysis including the results of cost per LYG, we conclude that trastuzumab treatment for IHC 3+ populations may be cost effective, whereas the cost effectiveness for treating IHC 2+/FISH+ or IHC 3+ is debatable. Using trastuzumab to treat the all HER2-positive populations is not likely to be cost effective and needs further investigation. We tested utility scores ranging from 0.4 (half of the first-line stage) to 0.8 (same as the first-line stage) in the sensitivity analysis. If the actual utility score decreases to 0.4, the ICER of trastuzumab for the IHC 3+ population is JPY 7.5 million (€68 000); if it increases to 0.8, the ICER for the entire HER2-positive population is JPY 10 million (€93 000). Accordingly, the results are similar.

Many economic evaluation studies have shown that trastuzumab for metastatic or early breast cancer is cost effective (McKeage and Lyseng-Williamson, 2008). The overexpression of HER2 is a poor-prognostic factor for breast cancer, but not for GC. This difference may reflect the additional benefits conferred by trastuzumab.

This study is fundamentally based on patient-level data obtained from the ToGA trial. By using actual trial data, we reduced assumptions about prognosis and resource utilisation that are often required in economic analyses. We used parametric survival analysis to extend the tails of the survival curve using a Weibull model (Lawless, 2003). Figure 2 shows the estimated survival curve fitted to the nonparametric Kaplan–Meier curves, which also provides evidence of the validity of our model. However, there are no available data on utility scores after progression, which could influence the results. Although we performed a sensitivity analysis to evaluate the influence of uncertain parameters on our results, this is a limitation of our research. In addition, we could not trace all the medical resource utilisation in the study. As improved PFS may have potential impact on resources used to manage disease or related complications, it may lead to reduce the need for other medical interventions.

Another limitation of our analysis is the potential heterogeneity of effectiveness and resource consumption between Asians and the international population. For instance, PFS and OS of Asian populations are longer compared with the whole population, and Asian patients receive second-line chemotherapy more frequently. However, we arrived at the same conclusion as NICE, that is, trastuzumab for the IHC 3+ population is cost effective, although comparators are different. This strengthens the generalisability of our results. Furthermore, trastuzumab for the entire HER2-positive population is unlikely to be cost effective in the general population because the prognosis of Asian populations is better than that of the whole population. As we do not have data on the effectiveness of trastuzumab combined with epirubicin plus cisplatin and capecitabine in large RCTs, further studies will be required to determine the cost effectiveness of the triplet regimen, which is used in Europe/America.

New monoclonal antibodies are rapidly being developed. These drugs tend to be expensive and are not necessarily cost effective. In some countries, access to expensive anti-cancer drugs is limited (Mason *et al*, 2010) because they are not considered cost effective. Our analysis also indicates that the cost effectiveness of therapies such as trastuzumab may well increase by restricting them to specific populations. Although many other molecular targeting drugs, especially for metastatic solid cancers, are considered to be not cost effective, it is important that our analysis can find cost-effective subgroup when advanced GC is treated by trastuzumab.

## Figures and Tables

**Table 1:** Patient demographics

	**Trastuzumab plus chemotherapy (%)**	**Chemotherapy alone (%)**
*Region*		
Japan	51 (45)	50 (45)
Korea	62 (55)	60 (55)
		
*Female*	22 (19)	26 (24)
		
*Capecitabine regimen*	113 (100)	110 (100)
		
*ECOG PS*		
0–1	112 (99)	108 (98)
2	1 (1)	2 (2)
		
*Primary tumour site*		
Stomach	110 (97)	104 (95)
GE junction	3 (3)	6 (5)
		
*Type of GC*		
Intestinal	81 (72)	82 (75)
Diffuse	9 (8)	7 (6)
Mixed	23 (20)	21 (19)
		
*Measurable tumour*	102 (90)	88 (80)
		
*Extent of disease*		
Locally advanced	1 (1)	3 (3)
Metastatic	112 (99)	107 (97)
Previous gastrectomy	35 (31)	26 (24)
		
*HER2 status*		
FISH +/IHC 0	6 (5)	19 (17)
FISH +/IHC 1+	17 (15)	12 (11)
FISH +/IHC 2+	37 (33)	23 (21)
FISH +/IHC 3+	44 (39)	50 (45)
FISH −/IHC 3+	3 (3)	1 (1)
FISH +/IHC no result	2 (2)	1 (1)
FISH no result/IHC 3+	4 (4)	4 (4)

Abbreviations: ECOG=Eastern Cooperative Oncology group; FISH=fluorescence *in situ* hybridisation; GC=gastric cancer; GE=gastroesophageal; HER2=human epidermal growth factor type-2; IHC=immunohistochemical; PS=performance status.

**Table 2:** Frequency of anti-cancer drugs used after progression

**Anti-cancer drugs**	**Percentage (%)**
Paclitaxel	83.2
Irinotecan	70.3
TS-1	51.5
Docetaxel	21.8
Fluorouracil	16.8
Cisplatin	15.8
Calcium levofolinate	5.9

**Table 3:** Results of base-case analysis

	**ICER(/QALY)**		**ICER(/LYG)**	
	**JPY**	**Euro**	**JPY**	**Euro**
All HER2-positive	12 180 000	110 000	8 910 000	81 000
IHC 2+/ FISH+ or IHC 3+	9 080 000	83 000	6 630 000	60 000
IHC 3+	6 070 000	55 000	4 290 000	39 000
				
*(a) All HER2-positive*				
	**C (JPY)**	**IC (JPY)**		
Trastuzumab plus chemotherapy	2 940 000 (€27 000)	1 630 000 (€15 000)		
Chemotherapy alone	1 320 000 (€12 000)	—		
				
	**E (QALY)**	**IE (QALY)**	**ICER (JPY/QALY)**	
Trastuzumab plus chemotherapy	1.168	0.134	1 218 000 (€110 000)	
Chemotherapy alone	1.048	—	—	
				
	**E (LY)**	**IE (LY)**	**ICER (JPY/LY)**	
Trastuzumab plus chemotherapy	1.671	0.183	8 910 000 (€81 000)	
Chemotherapy alone	1.489	—	—	
				
*(b) IHC 2+/FISH+ or IHC 3+*				
	**C (JPY)**	**IC (JPY)**		
Trastuzumab plus chemotherapy	3 070 000 (€28 000)	1 780 000 (€16 000)		
Chemotherapy alone	1 290 000 (€12 000)	—		
				
	**E (QALY)**	**IE (QALY)**	**ICER (JPY/QALY)**	
Trastuzumab plus chemotherapy	1.238	0.196	9 080 000 (€83 000)	
Chemotherapy alone	1.056	—	—	
				
	**E (LY)**	**IE (LY)**	**ICER (JPY10,000/LY)**	
Trastuzumab plus chemotherapy	1.764	0.268	6 630 000 (€60 000)	
Chemotherapy alone	1.495	—	—	
				
*(c) IHC 3+*				
	**C (JPY)**	**IC (JPY)**		
Trastuzumab plus chemotherapy	3 330 000 (€30 000)	1 980 000 (€18 000)		
Chemotherapy alone	1 350 000 (€12 000)	—		
				
	**E (QALY)**	**IE (QALY)**	**ICER (JPY/QALY)**	
Trastuzumab plus chemotherapy	1.371	0.326	6 070 000 (€55 000)	
Chemotherapy alone	1.060	—	—	
				
	**E (LY)**	**IE (LY)**	**ICER (JPY/LY)**	
Trastuzumab plus chemotherapy	1.935	0.462	4 290 000 (€39 000)	
Chemotherapy alone	1.473	—	—	

Abbreviations: C=cost; E=effectiveness; FISH=fluorescence *in situ* hybridisation; HER2=human epidermal growth factor type-2; IC=incremental cost; ICER=incremental cost-effectiveness ratio; IE=incremental effectiveness; IHC=immunohistochemical; QALY=quality-adjusted life year.

**Table 4:** Results of sensitivity analysis (ICER: JPY per QALY gained): (a) Utility scores after progression; (b) costs of AEs (additional costs of trastuzumab group)

	**All the HER2-positive**	**IHC 2+/ FISH +/ or IHC 3+**	**IHC 3+**
*(a) Utility*			
0.8	1 019 000 (€93 000)	7 750 000 (€70 000)	5 130 000 (€47 000)
0.7	1 110 000 (€101 000)	8 370 000 (€76 000)	5 560 000 (€51 000)
0.6	1 218 000 (€111 000)	9 080 000 (€83 000)	6 070 000 (€55 000)
0.5	1 351 000 (€123 000)	9 940 000 (€90 000)	6 690 000 (€61 000)
0.4	1 516 000 (€138 000)	1 096 000 (€100 000)	7 450 000 (€68 000)
			
*(b) AE costs*			
10 000	1 226 000 (€111 000)	9 140 000 (€83 000)	6 100 000 (€55 000)
30 000	1 241 000 (€113 000)	9 240 000 (€84 000)	6 160 000 (€56 000)
50 000	1 256 000 (€114 000)	9 340 000 (€85 000)	6 230 000 (€57 000)
100 000	1 293 000 (€118 000)	9 590 000 (€87 000)	6 380 000 (€58 000)

Abbreviations: AE=adverse event; FISH=fluorescence *in situ* hybridisation; HER2=human epidermal growth factor type-2; ICER=incremental cost-effectiveness ratio; IHC=immunohistochemical; QALY=quality-adjusted life year.
